# Chemical Composition and Geographic Variation of Cold Pressed *Balanites aegyptiaca* Kernel Oil

**DOI:** 10.3390/foods13071135

**Published:** 2024-04-08

**Authors:** Said El Harkaoui, Asma El Kaourat, Hanae El Monfalouti, Badr Eddine Kartah, Abdalbasit Adam Mariod, Zoubida Charrouf, Sascha Rohn, Stephan Drusch, Bertrand Matthäus

**Affiliations:** 1 Department for Safety and Quality of Cereals, Max Rubner-Institut, Federal Research Institute for Nutrition and Food, Schützenberg 12, 32756 Detmold, Germany; said.elharkaoui@mri.bund.de; 2 Department of Food Chemistry and Analysis, Institute of Food Technology and Food Chemistry, Technische Universität Berlin, Gustav-Meyer-Allee 25, 13355 Berlin, Germany; rohn@tu-berlin.de; 3 Department of Food Technology and Food Material Science, Institute of Food Technology and Food Chemistry, Technische Universität Berlin, Königin-Luise-Str. 22, 14195 Berlin, Germany; stephan.drusch@tu-berlin.de; 4 Laboratory of Plant Chemistry and Organic and Bio-Organic Synthesis, Faculty of Sciences, Mohammed V University in Rabat, 4 Avenue Ibn Battouta B.P., Rabat RP 1014, Morocco; asma.elkaourat@gmail.com (A.E.K.); h.elmonfalouti@um5r.ac.ma (H.E.M.); b.kartah@um5r.ac.ma (B.E.K.); zcharrouf@yahoo.fr (Z.C.); 5 Department of Biological Science, College of Science, University of Jeddah, Jeddah 21931, Saudi Arabia; basitmariod58@gmail.com; 6 Indigenous Knowledge and Heritage Center, Ghibaish College of Science & Technology, Ghibaish P.O. Box 100, Sudan

**Keywords:** *Balanites aegyptiaca*, date oil, fatty acid, triacylglycerol, tocochromanol, phytosterol, water deficit, temperature, multivariate analysis, sustainable utilization

## Abstract

With the increasing impacts of climate change, establishing more sustainable and robust plants such as desert dates (*Balanites aegyptiaca*) seems to be necessary. Known for its resilience in arid conditions, this tree has the potential to become a more important food source, particularly for its potential to yield edible oil. This study characterized *Balanites* kernel oil (BKO) as a promising oil source in arid regions, studying the influence of geographical origin and environmental factors. Moroccan and Sudanese BKO samples were analyzed and compared with Mauritanian BKO. In the fatty acid profile, unsaturated fatty acids constituted over 70% of the BKO profile, with a predominance of linoleic acid (Li), oleic acid (Ol), palmitic acid (Pa), and stearic acid (St). Consequently, the predominant triacylglycerols were PaLiLi, PaLiOl, LiLiOl, OlLiOl, and StLiOl. α-Tocopherol dominated the tocochromanol composition (324 to 607 mg/kg), followed by γ-tocopherol (120 to 226 mg/kg), constituting 90% of the total tocochromanols. The total phytosterol content in BKO ranged from 871 to 2218 mg/kg oil, with β-sitosterol dominating (58% to 74%). Principal Component Analysis revealed that the geographical origin significantly influences BKO composition, emphasizing environmental factors, particularly water deficit and/or temperatures. Notably, Moroccan BKO collected from an area characterized by high aridity and relatively low winter temperatures, showcased a unique profile in fatty acid, phytosterols, and tocochromanols. The valorization of BKO presents an opportunity for local agricultural development in arid regions and a role model for plant development and agricultural practices in other parts of the world.

## 1. Introduction

Establishing more sustainable and robust plants such as desert dates (*Balanites aegyptiaca* (L.) Delile.) seems to be necessary, because of the increasing impacts of climate change. This tree holds great esteem across the Sahelian and Saharan regions for its diverse traditionally uses, including its wood suitable for lighting fires for cooking, its edible fruits with a sweet mesocarp and the oil made from the kernels, its animal feed value, and its numerous ethnomedicinal applications [1,2].

**Figure 1:**
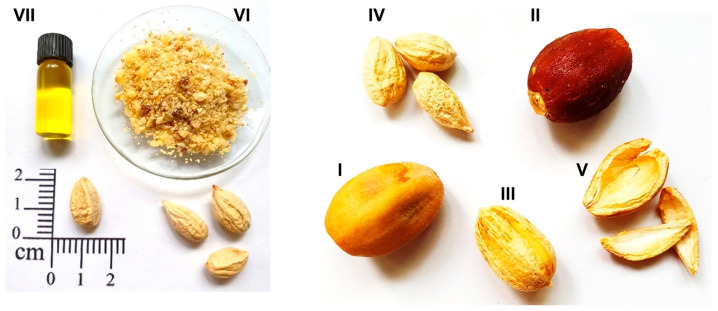
Different parts of *Balanites aegyptiaca* fruit and *Balanites aegyptiaca* kernel oil. I: mature fruit, II: pulp, III: nut, IV: kernel, V: nut shell, VI: milled kernel, VII: kernel oil.

*Balanites aegyptiaca* is a multibranched, spiny, evergreen wild shrub or tree that grows up to a height of 10 m [3]. It is a widely grown desert plant, native to arid and semi-arid areas of Africa, the Arabian Peninsula, and South Asia [3]. The plant is known by various names, such as the desert date tree in the English language or *Heglig*, *Zaqqum*, and *Lalob* in the Arabic language, and it is also referred to as *Tugga*, *Hlilaje*, or *Taberkat* in some parts of Mauritania and in the Moroccan Desert [4,5,6]. The annual fruit yield per tree can reach 100–150 kg [7]. The fruit, a plum-sized and faintly five-grooved drupe, consists of an epicarp (5–9%), the mesocarp (28–33%), the endocarp (49–54%), and the kernel (8–12%) [8]. The fruit is green in the first stages of growth and turns yellow to brownish when ripe; it weighs on average 5.7 g and the kernel is about 0.7 g. Different parts of the *Balanites* fruit are illustrated in Figure 1. The dark fleshy pulp, the edible part of the fruit, contains 1.2–1.5% protein and a high sugar content (35–42%), predominantly present as reducing sugars (81.3–91.1%) [4]. Moreover, the fruits are also rich in minerals, including calcium, magnesium, phosphorus, potassium, and sodium [9]. Particularly noteworthy is the kernel’s substantial oil content (40–51%), comprised of diverse fatty acids including saturated, mainly palmitic and stearic acid, and unsaturated, mainly oleic and linoleic acid, making it a potential source for various applications [1,10,11]. Traditionally used in African and Indian pharmacopeias for treating ailments such as jaundice, epilepsy, and rheumatism, BKO has particularly gained attention in biodiesel production, and many publications support this application [8,12,13,14,15,16]. However, the focus should be extended beyond the prevailing trend in biodiesel production, aiming to redirect research towards a promoting broader application of BKO particularly in the food or cosmetic sectors, positioning BKO as a promising source of vegetable oil, especially in arid regions, but also as a role model for plant development and agricultural practices in other parts of the world.

In recent years, noteworthy efforts have emerged, particularly in countries like Mauritania, where local cooperatives have taken the initiative to produce and commercialize BKO as both an edible oil (extracted from roasted kernels) and a cosmetic oil (extracted from unroasted kernels), drawing parallels with the production of argan oil in Morocco. This approach serves as a model that warrants encouragement and promotion in other regions where the *Balanites* tree exists. While local initiatives mark a positive stride, there exists a critical gap in comprehensive knowledge regarding the limitations in knowledge of its chemical composition, especially, how various factors like geographic origin and climate conditions, may impact composition.

The aim of this study was to provide additional insights into the lipid composition of BKO focusing on fatty acids, tocochromanols, phytosterols, and triacylglycerols and their variation according to geographical origin. This will contribute to introduce the *Balanites* tree to the scientific community as a potential source of edible vegetable oil. For the characterization, samples from Morocco and Sudan were included in this study and compared with BKO produced by a Mauritanian cooperative serving as a reference. Despite the relatively small number of samples, multivariate statistics were used to test whether there were differences in the composition of the oils with respect to the location of cultivation.

## 2. Materials and Methods

### 2.1. Material 

*Balanites* kernel oil, labeled as “alimentary” and “cosmetic”, was obtained from a local cooperative in *Guidimakha*, Mauritania, and used as a reference. Additionally, *Balanites* fruits were harvested at the maturity stage from Morocco (three samples collected from the *Tata* area over three successive harvest years: 2020, 2021, and 2022) and Sudan (collected from three areas: *El Fulah*, *Al Fashir*, and *Al ‘Abbasiyah*). The fruit processing steps included removing the pulp by soaking the entire fruit in warm water, washing it, drying the nuts in the sun, and finally cracking them to collect the kernels. These processing steps mirror those of the cooperatives in Mauritania. Details about the sample set, including corresponding temperatures and rainfall in the collection areas, can be found in Table 1. Additional information is available in Table S1.

### 2.2. Cold Pressing Process

Mechanical pressing of *B. aegyptiaca* kernels was carried out using a Komet single press (IBG Monforts Oekotec GmbH & Co. KG, Mönchengladbach, Germany). Due to the soft nature of kernels, small pieces of their shells were added in a 2:1 ratio (kernel: shell) to induce a friction effect during pressing. The pressing was performed using an 8 mm nozzle at temperatures kept below 60 °C, while the screw rotated at a speed of 35 rpm. The temperature of the resultant oil, remained below 40 °C. The extract obtained was centrifuged at 1550× *g* for 15 min to separate the oil from the sediment. All the oil samples were carefully preserved in sealed dark glass bottles and stored in a freezer (−18 °C) until later analysis.

### 2.3. Determination of the Fatty Acid Composition

The fatty acid composition was assessed through gas chromatography, employing the standard methodologies DGF C-VI 10a (00) and C-VI 11d (19) [18]. Initially, a droplet of oil was dissolved in 1 mL of *n*-heptane (LiChrosolv^®^, Merck KGaA, Darmstadt, Germany) for liquid chromatography. Subsequently, 50 μL of sodium methylate (30% solution in methanol; Merck KGaA, Darmstadt, Germany) were added and mixed with the oil. After brief agitation at room temperature for 1 min, 100 μL of Millipore water were introduced. The mixture was centrifuged at 1550× *g* for 5 min, and the lower aqueous phase was removed. Following this, 50 μL 1 M hydrochloric acid (min. 25%, p.a; Chemsolute^®^, Th.Geyer GmbH & Co. KG, Renningen, Germany) and a few drops of methyl orange indicator (ACS reagent, dye content 85%; Merck KGaA, Darmstadt, Germany) were added. After a brief mix, the lower aqueous phase was removed. Twenty milligrams of sodium hydrogen sulfate (EMSURE^®^ ACS, ISO, Reag. Ph Eur, Merck KGaA, Darmstadt, Germany) were added. After centrifugation at 1550× *g* for 5 min, the upper *n*-heptane phase was injected into an HP5890 gas chromatograph (Agilent Technologies Deutschland GmbH, Waldbronn, Germany). The chromatograph was equipped with a CP-Sil 88 capillary column (100 m × 0.25 mm × 0.25 µm; Agilent Technologies Deutschland GmbH, Waldbronn, Germany). The temperature was gradually increased from 150 °C to 250 °C at a rate of 1.5 °C/min and held at 250 °C for 5 min. The injector and detector temperatures were set to 260 °C, and the carrier gas (H_2_) flow rate was 1.7 mL/min with a 1:50 split ratio. The fatty acid methyl esters (FAME) were identified by comparing their retention times with a standard mix (Supelco^®^37 Component FAME Mix; Merck KGaA, Darmstadt, Germany), and their composition was quantified as a percentage of the total fatty acids (FA).

### 2.4. Determination of the Triacylglecerol Composition

The triacylglycerol (TAG) composition was evaluated using gas chromatography following the standard method DGF C-VI 14 (08) [18]. The analysis was conducted on an Agilent 6890 gas chromatograph equipped with a flame ionization detector (Agilent Technologies Deutschland GmbH, Waldbronn, Germany). Fifty milligrams of oil were dissolved in 10 mL isooctane (EMSURE^®^, ACS, Reag. Ph Eur; Merck KGaA, Darmstadt, Germany), and 1 μL of this solution was injected into a Restek™ RTX^®^65TG column (30 m × 0.25 mm × 0.1 µm; Restek Corp., Bellefonte, PA, USA). The oven temperature was maintained at 300 °C for 1 min, then ramped from 300 °C to 360 °C at a rate of 2 °C/min and held at 360 °C for 10 min. The injector and detector were set to 370 °C, and the carrier gas (H_2_) flow rate was 1 mL/min with a 1:40 split ratio. The detector operated using 40 mL/min hydrogen, 450 mL/min air, and 45 mL/min nitrogen. According to the DGF method, TAGs were identified by comparing their retention times with those of sesame oil, which were previously established for TAG composition analysis. The composition of the individual triacylglycerols was calculated as percentage of the individual peak areas from the total peak areas of all peaks.

### 2.5. Determination of the Tocochromanol Composition 

To determine the tocochromanol content and composition, the standard method DGF F-II 4a (00) [18] was used. Initially, 150 mg of oil was dissolved in 1 mL of n-heptane (for liquid chromatography, LiChrosolv^®^, Merck KGaA, Darmstadt, Germany) and underwent filtration using a 1.0 μm PTFE filter (Whatmann™, Cytiva, Buckinghamshire, UK) followed by a 0.45 μm PTFE filter (Restek Corp., Bellefonte, PA, USA). The resulting filtered solution was then transferred into a vial and subsequently injected into the HPLC-FLD. The HPLC system comprised a pump (L-7100 LaChrom Elite^®^, Merck KGaA, Darmstadt, Germany), an autosampler (Spark Holland BV, Emmen, The Netherlands), a fluorescence detector (L-2485 LaChrom Elite^®^, Merck KGaA, Darmstadt, Germany), and the interface 35900E (software CAG BootP Server, Agilent Technologies Inc., Santa Clara, CA, USA). A diol phase column (25 cm × 4 mm × 5 μm, LiChroCART^®^ 250-4, Merck KGaA, Darmstadt, Germany) was used for isocratic separation. The mobile phase consisted of n-heptane/tert.butyl methyl ether (96:4 (*v*/*v*), for liquid chromatography, LiChrosolv^®^, Merck KGaA, Darmstadt, Germany) at a flow rate of 1.3 mL/min. For all samples, the injection volume was 20 μL, and the analysis was conducted over 66 min. The fluorescence detector settings were an excitation wavelength of 295 nm and an emission wavelength of 330 nm. Identification of tocochromanols was done using α-, β-, δ-, and γ-tocopherol reference standards (chromatographic purity 97.6–99.6%, Merck KGaA, Darmstadt, Germany), and quantification was achieved through external calibration using standard solutions (0.25–40 µg/mL).

### 2.6. Determination of the Phytosterol Composition 

The phytosterol composition was determined following the standard method DGF F-III 1 (98) [18]. An internal standard, betulin (98%; Merck KGaA, Darmstadt, Germany), was dissolved in diisopropyl ether (EMSURE^®^ ACS, Reag. Ph Eur; Merck KGaA, Darmstadt, Germany) at 1 mg/mL concentration. Subsequently, 250 mg of oil underwent refluxing in 5 mL ethanolic KOH solution for 15 min after adding the internal standard. The unsaponifiable components were isolated using solid phase extraction on an aluminum oxide column (Aluminum Oxide 90, Merck KGaA, Darmstadt, Germany). A thin-layer chromatography (TLC silica gel 60 plates, 20 cm × 20 cm; Merck KGaA, Darmstadt, Germany) was then used to separate the sterol fraction from the unsaponifiable matter, employing a mobile phase of *n*-heptane (LiChrosolv^®^, Merck KGaA, Darmstadt, Germany) and distilled diethyl ether (min. 99.5%, stabilized with BHT, Chemsolute^®^, Th.Geyer, Renningen, Germany) in a 50:50 *v*/*v* ratio. After separation, the sterol fraction was converted into silylated derivatives (TMS) using a silylating agent (*N*-Methyl-*N*-trimethylsilyl-heptafluorobutyramid, Macherey-Nagel GmbH & Co. KG, Düren, Germany). Gas chromatography (HP6890N, Agilent Technologies Deutschland GmbH, Waldbronn, Germany) was employed to determine the sterol composition. Compounds were separated on an SE 54 CB column (50 mm × 0.25 mm × 0.1 µm; Macherey-Nagel GmbH & Co. KG, Düren, Germany) with the oven temperature increasing from 245 °C to 265 °C at a rate of 5 °C/min and maintained at 265 °C for 40 min. The injector and detector temperatures were set to 320 °C. Identification of substances was based on relative retention times compared to betulin. Additionally, standard compounds (cholesterol, 99%; campesterol, 65%; stigmasterol, 95%; β-sitosterol, 95%; Merck KGaA, Darmstadt, Germany) were applied in the identification process.

### 2.7. Statistical Analysis 

Chemical analyses were conducted in triplicates for each sample, with the mean calculated from these triplicates for subsequent analysis. Given the study’s nature and the limited sample size, assumptions regarding normality were considered. To assess statistical significance, a one-way ANOVA followed by a Tukey-Kramer HSD test (*p* < 0.05) was conducted using JMP software (JMP 14.3.0, SAS Institute Inc., Cary, NC, USA). Additionally, principal component analysis (PCA) was employed. Initially, the data were autoscaled, followed by PCA using JMP software for further insights into the relationships among variables.

## 3. Results and Discussion 

### 3.1. Fatty Acid Composition 

**Figure 2:**
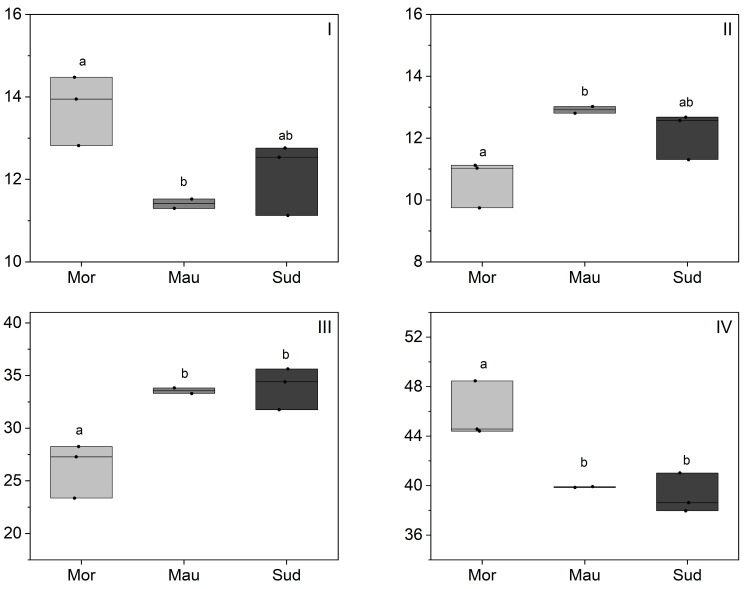
Fatty acid composition of BKO depending on geographic origin (%). Morocco (Mor), Mauritania (Mau), Sudan (Sud); (**I**) palmitic acid, (**II**) stearic acid, (**III**) oleic acid, (**IV**) linoleic acid. Statistical differences were identified using a one-way ANOVA test, which was then followed by a Tukey post-hoc test. Different letters indicated the existence of statistically significant differences with descriptive 95% confidence interval. Individual lines in box plots represent minimum, mean and maximum values.

The fatty acid (FA) composition of BKO samples was determined and the main FA are reported in Figure 2 (the full list of identified FA is given in Table S2). The unsaturated FA constitute over 70% of the FA composition with a predominance of linoleic acid (Li) at 38.0% to 48.5%, followed by oleic acid (Ol) at 23.4% to 35.6%. Palmitic (Pl) and Stearic (St) acids are present in comparable proportions, ranging from 11.1% to 14.5% and 9.7% to 13.0%, respectively. Additionally, minor amounts of α-linoleic acid, arachidic acid, vaccenic acid, and behenic acid were detected in the analyzed BKO samples. The abundance of linoleic acid, an essential omega-6 fatty acid, and oleic acid, a heart-healthy monounsaturated fatty acid, may promote BKO in food applications. In comparison to well established oils, the FA composition of BKO resembles that of sesame oil in terms of linoleic and oleic FA content [19,20]. FA composition is a critical factor in determining the oxidative stability and shelf life of vegetable oil, and vegetable oil with a low oleic/linolenic acid ratio, such as BKO, tends to have lower stability. Therefore, careful storage practices, including dark storage, nitrogen atmosphere, and refrigeration are recommended.

The FA composition of BKO has already been described partly in the literature with linoleic, oleic, palmitic, and stearic acids identified as the main FA. However, there are conflicting results regarding the predominant FA, with some studies agreeing with the present findings and indicating linoleic acid as the major FA, followed by oleic acid [3,8,14,21,22,23,24]. In contrast, other studies claimed that oleic acid is the most important FA in BKO, followed by linoleic acid [10,11,12,25]. Assuming consistent fruit maturity across studies, differences may be attributed to ecophysiological factors, partly influenced by geographic origin as well. On the other hand, even within the same geographical location, a notable variability in FA composition is evident among *Balanites aegyptiaca* trees, particularly in the concentrations of oleic and linoleic FA [3]. These authors correlated the variance in FA composition with fruit morphology, highlighting that the highest oleic/linoleic ratio is observed in fruits characterized by the lowest pulp percentage [3]. However, the authors further highlighted that linoleic acid consistently retains its status as the primary FA, while the oleic/linoleic ratio may shift in response to fruit morphology. Based on the findings described by Mehanni et al. [22], Mohamed et al. [11], and Elbadawi et al. [21], extraction methods, including enzymatic extraction, solvent extraction, and different pre-processing practices like a prior roasting or boiling of the kernels, have been shown not to significantly affect the FA composition of BKO. Generally, appropriate roasting seems to have only a minimal effect on the fatty acid composition of oils derived from roasted kernels [26]. However, suboptimal roasting conditions may lead to a slight decrease in the percentage of polyunsaturated fatty acids, potentially due to thermal degradation [26]. In addition, the influence of various extraction techniques on the fatty acid composition appears to be minor [27,28]. Nevertheless, it remains essential to assess the effects of the mentioned pre-processing practices in terms of storability and oxidative stability considerations.

Regarding the geographical origin effects, significant differences in FA composition were observed among the analyzed BKO samples from Morocco, Sudan, and Mauritania, particularly in the main fatty acids, linoleic, oleic, palmitic, and stearic acid (Figure 2). Moroccan samples exhibited a distinctive profile characterized by higher levels of palmitic and linoleic acids and lower levels of stearic and oleic acids compared to samples from Mauritania and Sudan. This unique BKO profile in Morocco is likely attributed to the climate, marked by lower rainfall (water deficit) and a broader temperature range compared to Sudan and Mauritania (Table 1), suggesting an adaptive response to water deficit and/or temperature. The adaptability to water deficit and/or temperature changes associated with alterations in FA composition, is well-documented for several plant species and has often been linked to changes in enzymatic activities involved in FA biosynthesis [29,30,31]. The proposed biosynthetic pathway of FA underscores a reciprocal quantitative relationship among different FA, monitored by various enzymes responsible for transitions between FA, primarily ketoacyl synthase and fatty acid desaturase [30]. Within this biosynthesis pathway, lower temperatures may increase the activity of oleate desaturase, favoring the synthesis of linoleic acid [30,32], as reported for cottonseed, sunflower, and camelina oil [30]. This mechanism could also explain the higher levels of linoleic acid observed in Moroccan BKO samples, and with larger samples set more concrete assumptions should be made. However, understanding the complete behavior of the plant under wild conditions remains challenging due to the multitude of interacting factors such as soil type, salinity, day length, and solar radiation levels.

### 3.2. Triacylglycerol Composition

**Figure 3:**
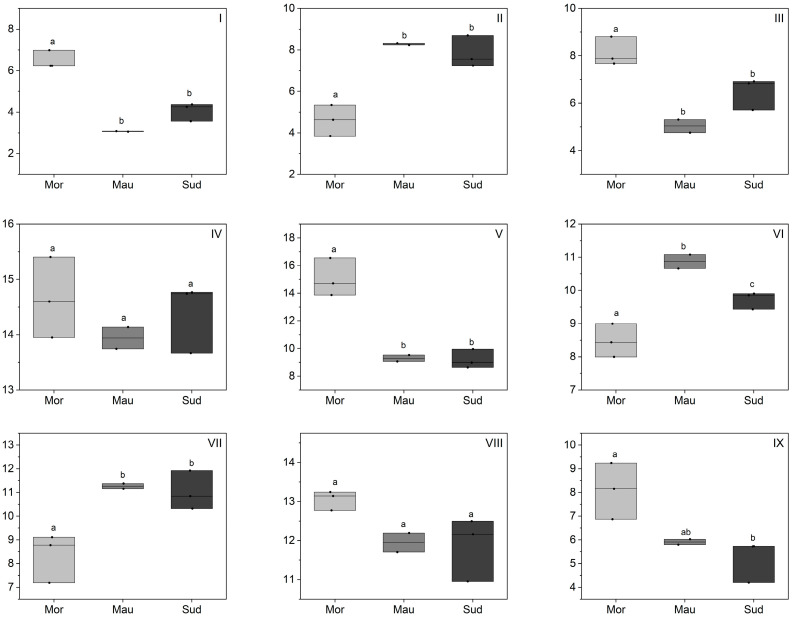
Triacylglycerol composition of BKO depending on the geographical origin (%). Morocco (Mor), Mauritania (Mau), Sudan (Sud); (**I**) PaLiPa, (**II**) PaOlOl, (**III**) PaLiSt, (**IV**) PaLiOl, (**V**) PaLiLi, (**VI**) StLiOl, (**VII**) OlLiOl, (**VIII**) LiLiOl, (**IX**) LiLiLi. Statistical differences were identified using a one-way ANOVA test, which was then followed by a Tukey post-hoc test. Different letters indicated the existence of statistically significant differences with a 95% confidence interval. Individual lines in box plots represent minimum, mean and maximum values.

A total of 14 triacylglycerols (TAG) were determined in BKO (Figure 3 and Table S3) with carbon numbers (CN) of 50, 52, and 54. The main TAG in the present BKO samples were PaLiLi (8.6–16.5%), PaLiOl (13.7–15.4%), LiLiOl (11.0–13.2%), OlLiOl (7.2–11.9%), and StLiOl (8–11.1%).

The TAG composition was composed of a mixture of different fatty acids, reflecting the relative concentrations of oleic, linoleic, palmitic, and stearic acids as the main fatty acids. In terms of predominance according to the carbon number in BKO TAG, the predominant ones had a carbon number of 54 (LiLiOl, OlLiOl, StLiOl, LiLiLi, OlOlOl, StOlOl, StOlSt), followed by 52 (PaLiLi, PaLiOl, PaLiSt, PaOlOl), and then 50 (PaLiPa, PaLiPa, PaOlPa). The study described by Diedhiou et al. [24] reported only three TAG in BKO (PaPlLi, PaOlLi, and StOlLi). BKO contains the most commonly represented TAG among the various oils reported in the literature. PaLiPa, PaLiLi, PaOlOl; LiLiLi, OlLiLi were identified in over 90% of all edible vegetable oils reported in the literature [33,34].

The variation of the TAG composition according to geographical origin followed a trend similar to what was observed for the fatty acid composition. Thus, TAG containing linoleate or palmitate (PaLiLi, LiLiOl, PaLiPa, LiLiLi) were significantly higher in Moroccan samples, whereas TAG containing oleate and stearate (StOlOl, StLiOl, PaOlOl, PaOlSt) were lower in Moroccan BKO samples compared with the samples from Sudan and Mauritania. Moroccan samples were characterized by a high level of TAG with CN 52 for TAG assembly. Thus, water deficit and/or temperature could have resulted in a different balance of isoforms for the lysophosphatidate acyltransferase or diacylglycerol acyltransferase reactions used in TAG accumulation. This hypothesis was suggested for differences in TAG for olive oil; however, no definite enzymatic evidence on these possibilities was suggested [35].

The presence of common TAG in BKO, challenges the establishment of a distinct TAG fingerprint; however, quantitative differences compared with other oils may serve as indicators.

### 3.3. Tocochromanol Composition 

**Figure 4:**
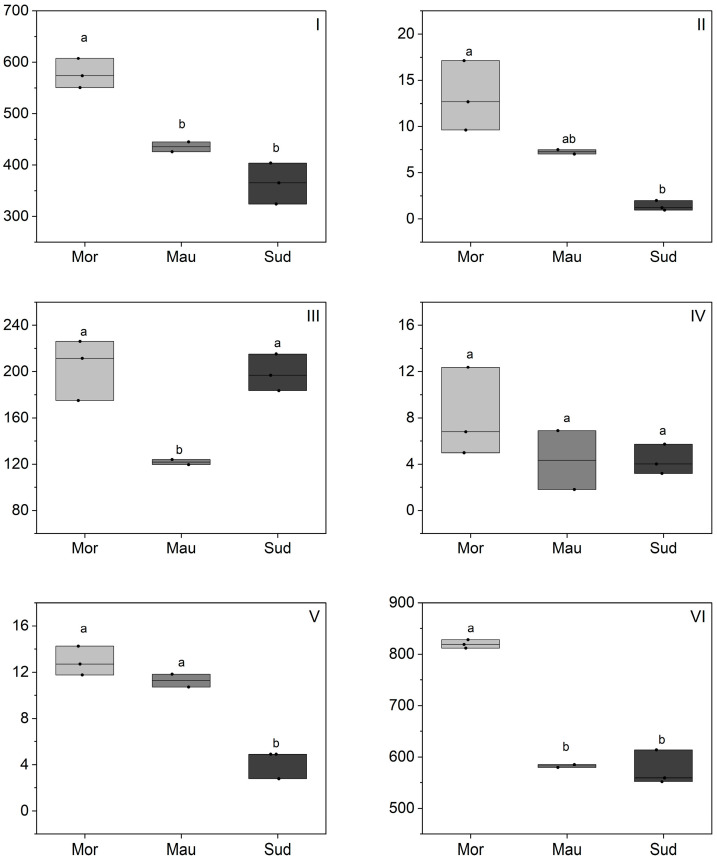
Tocochromanol composition of BKO depending on geographic origin (mg/kg). Morocco (Mor), Mauritania (Mau), Sudan (Sud); (**I**) α tocopherol, (**II**) β-tocopherol, (**III**) γ-tocopherol, (**IV**) plastochromanol-8, (**V**) δ-tocopherol, (**VI**) total tocochromanols. Statistical differences were identified using a one-way ANOVA test, which was then followed by a Tukey post-hoc test. Different letters indicated the existence of statistically significant differences with a 95% confidence interval. Individual lines in box plots represent minimum, mean and maximum values.

The tocochromanol composition of BKO was determined and showed total contents between 552 mg/kg and 828 mg/kg (Figure 4 and Table S4). Notably, α-tocopherol (α-toc) predominated in all analyzed samples, ranging from 324 to 607 mg/kg, followed by γ-tocopherol (γ-toc) ranging from 120 to 226 mg/kg, both constituting 90% of the total tocochromanol composition. Only two studies have been reported so far on the tocochromanol composition of BKO, indicating total tocochromanol contents of 510 mg/kg for BKO from Senegal [24] and 420 mg/kg for BKO from Sudan [21]. Both studies reported α-toc and γ-toc as the primary tocochromanols in BKO samples. The total tocochromanol content from these published studies was comparable to the content found in the present BKO from Mauritania and Sudan but lower than that found in the Moroccan BKO. In a study described by Elbadawi et al. [21], they also evaluated the effect of roasting or boiling *Balanites* kernels before oil extraction (part of the household extraction of BKO in some areas) on tocochromanol composition and reported no significant differences. In comparison to other oils, BKO is characterized by a higher tocochromanol content than olive oil and comparable values to argan and cactus oil; the particularity of BKO lies in its high content of α-toc, unlike argan and cactus oil, which are rich in γ-toc [27,36,37,38]. The high content of α-toc in BKO can be regarded as advantageous as it is considered the most biologically active form of vitamin E, which is preferentially utilized by the human body [39]. 

Regarding the geographical origin effect on the tocochromanol composition of BKO, significant differences were found. Moroccan samples exhibited a higher amount of total tocochromanol (mean value: 819 mg/kg) compared to Mauritania (mean 582 mg/kg) and Sudan (mean 575 mg/kg). Across all geographical origins, α-toc was the primary isomer. Moroccan samples displayed a high α-toc content (mean 577 mg/kg) in contrast to Mauritania (mean 435 mg/kg) and Sudan (mean 364 mg/kg). The elevated tocochromanol levels in BKO from Morocco could be attributed to the effects of water deficit, aligning with findings in other oils such as almond, olive, and soybean [40,41,42]. Additionally, cold stress may also contribute to the elevated tocochromanol levels in Moroccan BKO, as reported for rice [43]. The authors conducted expression profiling studies, demonstrating that cold stress enhances the expression of p-hydroxyphenylpyruvate dioxygenase (HPPD), leading to an increased supply of precursors for the biosynthesis of tocochromanols [43]. This first aspect of variation underscores the influence of ecophysiological factors on the overall tocochromanol profile. For a second aspect, the percentage of individual tocochromanol, it was noted that the percentage of each isomer to the total tocochromanol content varies based on geographical origin. The percentage of α-toc in Moroccan and Mauritanian samples was 70% (mean value) and 75% (mean value), respectively, while in Sudan, it represented only 63% (mean value). Conversely, the percentage of γ-toc in Moroccan and Mauritanian samples was 25% (mean value) and 21% (mean value), respectively, whereas in Sudan, γ-toc represented 35%. This variation in α- and γ-toc between analyzed BKO samples may be explained by the biosynthesis pathway influenced by water deficit and/or temperature, which differed in the collection areas (Table 1). The biosynthesis pathway of tocopherol (shikimate pathway), starting with p-hydroxyphenylpyruvic (HPP), shows that there is a relationship between α- and γ-toc, as α-toc is generated from the methylation of the aromatic ring of γ-toc, and this reaction is catalyzed by γ-toc methyl-transferase (γ-TMT) [44]. The higher α-toc/γ-toc in Moroccan and Mauritanian BKOs may be due to a water deficit and/or temperature, catalytic effect on γ-TMT. A similar trend of variation was also found in other plant species [40,41,45]. 

### 3.4. Phytosterol Composition 

**Figure 5:**
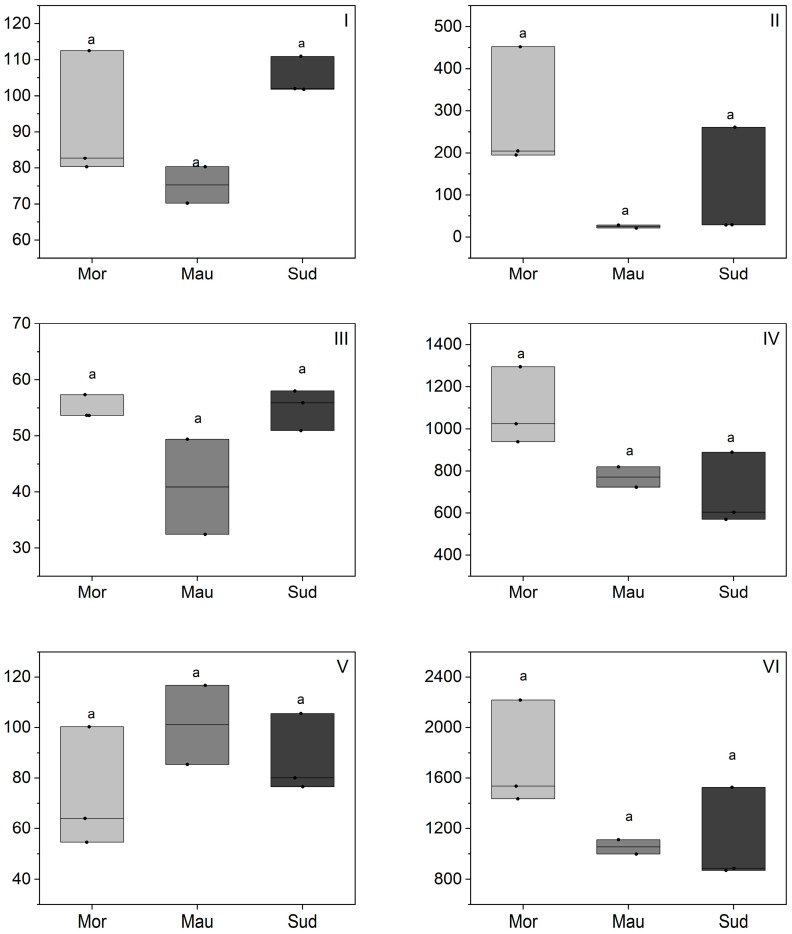
Phytosterol composition of BKO depending on geographic origin (mg/kg). Morocco (Mor), Mauritania (Mau), Sudan (Sud); (**I**) cholesterol, (**II**) campesterol, (**III**) stigmasterol, (**IV**) β-sitosterol, (**V**) Δ5-avenasterol, (**VI**) total phytosterol. Statistical differences were identified using a one-way ANOVA test, which was then followed by a Tukey post-hoc test. Different letters indicated the existence of statistically significant differences with a 95% confidence interval. Individual lines in box plots represent minimum, mean and maximum values.

The phytosterol composition of BKO is detailed in Figure 5 and Table S5, revealing a comprehensive profile. The total phytosterol content in BKO ranged from 871 to 2218 mg/kg oil, encompassing a diverse mixture of phytosterols. Notably, β-sitosterol dominates across all analyzed samples, with concentrations ranging from 570 to 1295 mg/kg oil, constituting 58% to 74% of the total phytosterol content in BKO. The results align with comparative studies in Senegal, where the reported total phytosterol content is 2110 mg/kg oil, and Nigeria, where it is 1102 mg/kg, emphasizing also the prevalence of β-sitosterol in BKO [24,46]. However, these were the only studies reporting a phytosterol composition of BKO. In comparison with other edible oils from arid regions like argan kernel oil or cactus seed oil, BKO exhibits comparable phytosterol content to argan oil and lower content in comparison with cactus oil [27,36,37]. The ubiquitous dominance of β-sitosterol in various vegetable oils, including soybean, corn, sesame, and palm oils is well-documented [19]. β-Sitosterol has multiple applications in various fields such as medicine and the global food industry [47] and this may promote BKO in food applications as well. In addition to β-sitosterol, BKO showcases other common phytosterols such as campesterol, Δ5-avenasterol, and stigmasterol. Cholesterol, previously detected in other vegetable oils such as camelina oil [48], was also found in the present BKO.

Regarding the geographical origin effect on the total phytosterol composition, the results revealed notable differences, with Moroccan samples exhibiting the highest total phytosterol content (mean 1730 mg/kg oil) compared to Mauritania (mean 1054 mg/kg oil) and Sudan (mean 1095 mg/kg oil). This discrepancy aligns with ecophysiological factors, particularly the severity of the water deficit in Moroccan regions (Table 1), offering a plausible explanation for the elevated phytosterol content in Moroccan samples. The trends observed are in line with findings reported on other oils. For instance, an investigation into olive oil documented a substantial decrease in total phytosterol content following irrigation-induced alterations, dropping from 1604 to 1005 mg/kg [35]. Similarly, a study describing the effect of water deficit on the total phytosterol composition of sunflower oil under managed and controlled field conditions reported an increase in total phytosterol content under water deficit conditions [49]. A parallel trend was also reported for rice [50], where higher total phytosterol contents were noted in response to water deficit, with contents rising proportionally to the number of days of water stress. The authors attributed this to the increasing expression of HMG-CoA reductase, the limiting enzyme in the biosynthesis of plant sterols [50]. On the other hand, cold stress was also reported to increase the level of phytosterols in different plants [51]. These insights could be extended to other plant species, including *Balanites*, although further studies are needed for confirmation. Regarding the variation in the proportions of individual phytosterols, no clear trend emerged for the present BKO samples. Literature shows mixed findings, with some studies indicating that phytosterol variations in response to water deficit and/or temperature effect result only in total phytosterol content variation, with very small changes in the percentages of each individual phytosterol [35,52]. In contrast, other studies reported changes in total phytosterols and the percentages of each individual phytosterol [49,53]. For instance, the total phytosterol content in soybean seeds was affected by the temperature in the growing location, while the phytosterol composition was almost the same in all soybean seed samples, and it was not affected by growing location temperature [52]. Similarly, in the case of olive oil, irrigation caused a significant decrease in total phytosterol content as described above, while only small changes in the percentages of each individual phytosterol were noted [35]. Conversely, in sunflower seeds, a delay in sowing, leading to higher temperatures during seed formation, induced a general increase in total sterol concentration by up to 35%, as well as a significant variation in the β-sitosterol/campesterol ratio [53].

### 3.5. Multivariate Analysis 

Principal component analysis (PCA) was employed to investigate the relationship between geographic location and chemical composition, providing initial insights into how the geographical origin influences the composition of BKO. This approach aimed to discern trends or correlations associated with specific origins by utilizing PCA, which was executed on a fused matrix comprising 8 rows corresponding to BKO samples and 44 columns representing the compositions of fatty acids, triacylglycerols, tocochromanols, and phytosterols. The combination of all variables into a single matrix allowed for the assessment of the contribution of both minor and major variables on a normalized scale.

**Figure 6:**
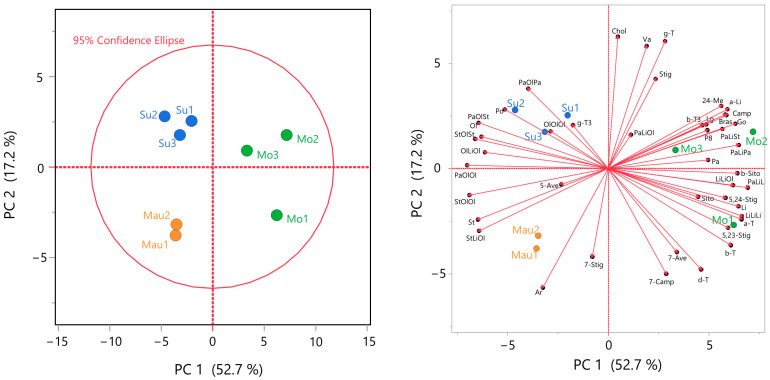
Score plot (**left**) and Biplot (**right**) resulting from Principal Component Analysis (PCA) based on the chemical composition of the examined BKOs. Variables ≤ 0.1 were not included in the multivariate analysis, behenic acid also removed since it has constant values. Morocco (Mor), Mauritania (Mau), Sudan (Sud), Pa: palmitic acid, Po: palmitoleic acid, St: stearic acid, Ol: oleic acid, Va: vaccenic acid, Li: linoleic acid, a-Li: α-linolenic acid, Ar: arachidic acid, Go: gondoic acid, Lg: lignoceric acid, a-T: α-tocopherol, b-T: β-tocopherol, g-T: γ tocopherol, b-T3: β-tocotrienol, P8: plastochromanol-8, g-T3: γ-tocotrienol, d-T: δ-tocopherol, Chol: cholesterol, Bras: brassicasterol, 24-Me: 24-methylenecholesterol, Camp: campesterol, Stig: stigmasterol, 7-Camp: Δ7-campesterol, 5,23-Stig: Δ5,23-stigmastadienol, b-Sito: β-sitosterol, Sito: sitostanol, 5-Ave: Δ5-avenasterol, 5,24-Stig: Δ5,24-stigmastadienol, 7-Stig: Δ7-stigmastenol, 7-Ave: Δ7-avenasterol. The full sample names are available in Table S1 for reference.

As shown in Figure 6 (left, PCA Score Plot), the first two principal components (PC) explained 70% of the total variance (PC1: 52.7%; PC2: 17.2%). The score plot revealed a distinct distribution of oils based on geographical origin, with Moroccan samples notably separated along PC1, and Mauritanian and Sudanese samples distinguished along PC2 (refer to Figure 6 on the left). To better understand and visualize the correlation between chemical composition and geographical origin, a biplot was generated (Figure 6, right, biplot). The biplot visually illustrates the correlation between variables and geographical origin, offering valuable insights into the chemical composition of BKO associated with different regions. The correlated variables with PCs encompassed various chemical classes, including different FA, TAG, tocochromanols, and phytosterols, signifying the influence of geographical origin on the entire analyzed chemical profile. Moroccan samples, situated in the positive part of PC1 (which exhibited maximum variance), displayed a distinct profile compared to Sudanese and Mauritanian samples. They exhibited positive correlations with Li and Pa fatty acids and corresponding TAG such as PaLiLi, LiLiLi, and PaLiPa. Additionally, positive correlations with α-tocopherol and β-sitosterol were also observed. The discrimination of Sudanese and Mauritanian samples occurred along the PC2 direction, with Sudanese samples in the positive part and Mauritanian samples in the negative part. The contribution of each variable is detailed in Figure S1, representing different loadings of the constructed PCA model.

The PCA results confirm the discussion in the preceding sections, suggesting that specific climate conditions, particularly water deficit and/or temperature, may explain the observed differences in the chemical composition of BKO samples. However, understanding the complete behavior of the plant under wild conditions remains challenging due to the multitude of interacting factors such as soil type, salinity, day length, and solar radiation levels.

## 4. Conclusions

The present study aimed at providing further insights into the lipid composition of BKO, focusing on FA, tocochromanols, phytosterols, and TAG and their variations based on geographical origin. Chemical and principal component analyses revealed significant geographical variation emphasizing the influence of ecosphysiological factors, notably water deficits and/or temperatures. While this study provides valuable insights into BKO’s chemical composition and its geographical variations, further research with a larger and more diverse sample set is strongly recommended for a comprehensive understanding. Future investigations will include the analysis of additional chemical compounds, especially polyphenols, and address the storability and oxidative stability, as well as the sensory properties of BKO. Setting and analyzing quality criteria that match legal aspects and being comparable to parameters that are already applied for other edible oils will help to make BKO a marketable edible oil. Despite its promising oil yield and chemical composition, the practicality of large-scale BKO production presents challenges, specifically with regards to the efficient removal of pulp and the cracking of *Balanites* nuts for kernel extraction. To ensure the high quality of the kernel, the utilization of appropriate machinery in the pulp removal process, discouraging the traditional household practice involving water is suggested. Additionally, for the cracking of nuts, the maintenance of manual cracking processes within local cooperatives is recommended. This approach, not only ensures efficient processing, but also guarantees an income for the local population and corresponds to the successful model of argan oil production, where the fruit is harvested and shelled manually while the subsequent steps are automated. The valorization of BKO might serve to motivate local communities to cultivate more resilient *Balanites* trees, especially in arid regions, thereby, promoting sustainability and fostering local agricultural development.

## Figures and Tables

**Table 1:** Harvest location, temperatures and rainfall. The rainfall and temperature data for the corresponding harvest year and location were obtained from Google Climate Engine [17].

Sample Code	Harvest Location	Temperature Min–Max (°C)	Rainfall (mm/Year)
Mo1	*Tata*–Morocco	3.98–41.04	69.7
Mo2	*Tata*–Morocco	6.08–41.54	64.0
Mo3	*Tata*–Morocco	6.02–41.49	205.4
Mau1	*Guidimakha*–Mauritania	18.03–43.49	352.5
Mau2	*Guidimakha*–Mauritania	18.03–43.49	352.5
Su1	*El Fulah*–Sudan	11.03–39.90	533.2
Su2	*Al Fashir*–Sudan	16.76–40.67	466.6
Su3	*Al ‘Abbasiyah*–Sudan	18.91–38.12	591.8

## Data Availability

The original contributions presented in the study are included in the article/Supplementary Material, further inquiries can be directed to the corresponding author.

## Supplementary Materials

The following supporting information can be downloaded at: https://www.mdpi.com/article/10.3390/foods13071135/s1, Table S1: Additional information on sample set, Table S2: Fatty acid composition of *Balanites* kernel oil (%), Table S3: Triacylglycerol composition of *Balanites* kernel oil (%), Table S4: Tocochromanol composition of *Balanites* kernel oil (mg/kg of oil), Table S5: Phytosterol composition of *Balanites* kernel oil (mg/kg of oil), Figure S1: Loadings of the two dimensions principal component analysis.
