# Supplementary material for: Chemical Composition and Geographic Variation of Cold Pressed Balanites aegyptiaca Kernel Oil
Source: Foods. 2024 Apr 8;13(7):1135. doi: 10.3390/foods13071135 (PMC11011647; doi:10.3390/foods13071135)
Supplement: Supplementary file 1 [file foods-13-01135-s001.zip › foods-2945624-supplementary.pdf]

Table S1. Additional information on sample set

Sample Code	Harvest location	Description
Mo1	*Tata* - Morocco	*Balanites* fruits were collected From *Tata* (Morocco) in 2020 and the extraction was done as described in the method part
Mo2	*Tata* - Morocco	*Balanites* fruit were collected From *Tata* (Morocco) in 2021 and the extraction was done as described in the method part
Mo3	*Tata* – Morocco	*Balanites* fruit were collected From *Tata* (Morocco) in 2022 and the extraction was done as described in the method part
Mau1	*Guidimakha* – Mauritania	Cosmetic *Balanites* kernel oil (extracted from non-roosted kernels) was purchased from a local cooperative in *Guidimakha*, in Mauritania
Mau2	*Guidimakha* – Mauritania	Alimentary *Balanites* kernel oil (extracted from kernels roasted for 10 min at 100 °C in the oven) was purchased from a local cooperative in *Guidimakha*, in Mauritania
Su1	*El Fulah* – Sudan	*Balanites* fruits were collected From *El Fulah* (Sudan) in 2021 and the extraction was done as described in the method part
Su2	*Al Fashir* – Sudan	*Balanites* fruits were collected From *Al Fashir* (Sudan) in 2021 and the extraction was done as described in the method part
Su3	*Al ′Abbasiyah* – Sudan	*Balanites* fruits were collected From *Al ′Abbasiyah* (Sudan) in 2021 and the extraction was done as described in the method part

Table S2. Fatty acid composition of *Balanites* kernel oil (%)

Sample	Mo1	Mo2	Mo3	Mau1	Mau2	Su1	Su2	Su3
**14:0**	0.1±0.0	0.1±0.0	nd	nd	nd	nd	0.1±0.0	0.1±0.0
**16:0**	14.5±0.5	12.8±0.1	13.9±0.4	11.5±0.3	11.3±0.2	11.1±0.1	12.8±0.1	12.5±0.1
**16:1 Δ9**	0.1±0.0	0.1±0.0	nd	nd	nd	nd	0.1±0.0	0.1±0.0
**17:0**	0.1±0.0	0.1±0.0	0.1±0.0	0.1±0.0	0.1±0.0	0.1±0.0	0.1±0.0	0.1±0.0
**18:0**	11.1±0.2	9.7±0.0	11.0±0.1	13.0±0.1	12.8±0.1	11.3±0.0	12.7±0.0	12.6±0.0
**18:1Δ9**	23.4±0.1	28.3±0.1	27.3±0.0	33.3±0.0	33.8±0.0	35.6±0.0	34.4±0.1	31.7±0.1
**18:1Δ11**	0.8±0.0	1.2±0.0	1.0±0.0	0.8±0.0	0.8±0.0	1.2±0.0	1.0±0.0	0.9±0.0
**18:2Δ9t,12 t**	nd	0.1±0.0	0.1±0.0	0.1±0.0	0.1±0.0	nd	nd	nd
**18:2Δ9,12**	48.5±0.3	44.4±0.1	44.6±0.1	39.9±0.2	39.8±0.1	38.6±0.0	38.0±0.1	41.0±0.0
**18:3Δ9,12,15**	0.7±0.0	1.7±0.0	0.8±0.0	nd	nd	0.9±0.0	0.1±0.0	0.1±0.0
**20:0**	0.4±0.0	0.4±0.0	0.4±0.0	0.5±0.0	0.5±0.0	0.4±0.0	0.4±0.0	0.4±0.0
**20:1Δ11**	0.2±0.0	0.3±0.0	0.2±0.0	0.1±0.0	0.1±0.0	0.2±0.0	0.1±0.0	0.1±0.0
**22:0**	0.1±0.0	0.1±0.0	0.1±0.0	0.1±0.0	0.1±0.0	0.1±0.0	0.1±0.0	0.1±0.0
**24:0**	0.1±0.0	0.5±0.0	0.2±0.0	0.1±0.0	0.1±0.0	0.1±0.0	0.1±0.0	0.1±0.0

Results are expressed as mean ± SD (n = 3), nd: not detected

Table S3. Triacylglycerol composition of *Balanites* kernel oil (%)

Sample	Mo1	Mo2	Mo3	Mau1	Mau2	Su1	Su2	Su3
**PaOlPa**	2.4±0.0	2.3±0.0	2.8±0.0	2.4±0.0	2.6±0.0	2.6±0.0	3.2±0.0	2.8±0.0
**PaLiPa**	7.0±0.0	6.2±0.0	6.2±0.0	3.1±0.0	3.1±0.0	3.6±0.0	4.3±0.0	4.4±0.0
**PaOlSt**	2.7±0.0	2.5±0.0	3.1±0.0	3.3±0.0	4.1±0.0	4.0±0.0	4.5±0.1	4.4±0.0
**PaOlOl**	3.8±0.0	4.6±0.0	5.3±0.0	8.2±0.0	8.3±0.0	7.6±0.0	8.7±0.1	7.2±0.0
**PaLiSt**	8.8±0.0	7.7±0.0	7.9±0.0	4.8±0.0	5.3±0.0	5.7±0.0	6.8±0.1	6.9±0.0
**PaLiOl**	14.6±0.0	13.9±0.0	15.4±0.0	14.1±0.0	13.7±0.0	13.7±0.1	14.7±0.1	14.8±0.1
**PaLiLi**	16.5±0.0	14.7±0.0	13.9±0.0	9.5±0.0	9.1±0.0	9.0±0.1	8.6±0.1	9.9±0.1
**StOlSt**	0.8±0.0	0.7±0.0	0.9±0.0	1.3±0.0	1.8±0.0	1.8±0.1	1.8±0.1	1.8±0.1
**StOlOl**	1.9±0.0	2.1±0.0	2.6±0.0	6.3±0.0	6.2±0.0	5.2±0.1	5.4±0.1	4.9±0.1
**OlOlOl**	3.3±0.0	6.8±0.0	4.5±0.0	6.3±0.0	6.5±0.0	7.5±0.0	6.1±0.1	4.8±0.1
**StLiOl**	8.4±0.0	8.0±0.0	9±0.0	11.1±0.0	10.7±0.0	9.4±0.1	9.9±0.1	9.9±0.1
**OlLiOl**	7.2±0.0	9.1±0.0	8.8±0.0	11.4±0.0	11.2±0.0	11.9±0.0	10.8±0.0	10.3±0.0
**LiLiOl**	13.2±0.0	13.1±0.0	12.8±0.0	12.2±0.0	11.7±0.0	12.5±0.0	11.0±0.0	12.2±0.0
**LiLiLi**	9.2±0.0	8.2±0.0	6.9±0.0	6.0±0.0	5.8±0.0	5.7±0.0	4.2±0.0	5.7±0.0

Results are expressed as mean ± SD (n = 3)

Pa: palmitic acid, St: searic acid, Ol: oleic acid, Li: linoleic acid

Table S4. Tocochromanol composition of *Balanites* kernel oil (mg/kg of oil)

	Mo1	Mo2	Mo3	Mau1	Mau2	Su1	Su2	Su3
**α-tocopherol**	607 ± 20	574 ± 2	551 ± 7	445 ± 8	426 ± 5	365 ± 9	324 ± 7	404 ± 17
**β-tocopherol**	17±1	13±0	10±0	7±0	7±0	1±0 <LOQ	1±0 <LOQ	2±0
**γ-tocopherol**	175±4	212±1	226±2	120±0	124±3	183±1	215±1	197±4
**β-tocotrienol**	nd	3±1	4±1	nd	nd	nd	nd	nd
**plastochromanol-8**	5 ± 0	12 ± 0	7 ± 0	2 ± 0	7 ± 1	6 ± 0	3 ± 1	4 ± 0
**γ-tocotrienol**	nd	3 ± 0	2 ± 0	1±0 <LOQ	4 ± 0	1±0 <LOQ	3 ± 0	2 ± 0
**δ-tocopherol**	14±1	12±1	13±0	11±1	12±0	3±0	5±0	5±1
**Sum**	819 ± 26	828 ± 5	812 ± 9	585 ± 8	580 ± 3	559 ± 10	552 ± 6	614 ± 22

Results are expressed as mean ± SD (n = 3), nd: not detected, LOQ: limit of quantification

Table S5. Phytosterol composition of *Balanites* kernel oil (mg/kg of oil)

Serols	Mo1	Mo2	Mo3	Mau1	Mau2	Su1	Su2	Su3
**Cholesterol**	82.6 ± 2.5	112.4 ± 22.1	80.3 ± 1.8	70.2 ± 0.2	77.9 ± 0.9	101.8 ± 1.6	110.9 ± 0.2	102.0 ± 1.0
**Brassicasterol**	51.9 ± 5.6	131.1 ± 0.6	61.9 ± 1.3	nd	nd	72.0 ± 4.1	nd	nd
**24-Methylenecholesterol**	13.0 ± 0.2	30.8 ± 0.8	14.0 ± 0.6	1.9 ± 0.1	nd	19.2 ± 0.2	2.8 ± 0.2	2.8 ± 0.0
**Campesterol**	194.9 ± 2.7	452 ± 0.9	204.6 ± 2.6	21.0 ± 0.2	28.1 ± 2.2	260.8 ± 1.9	28.6 ± 0.6	28.5 ± 0.3
**Stigmasterol**	57.3 ± 1.5	53.6 ± 4.7	53.6 ± 1.9	32.5 ± 2.2	49.4 ± 0.7	50.9 ± 1	58.0 ± 0.8	55.9 ± 0.6
**Δ7-Campesterol**	7.4 ± 0.6	5.0 ± 0.1	5.4 ± 0.3	4.8 ± 0.1	7.3 ± 0.6	4.2 ± 0.9	4.2 ± 0.3	3.0 ± 0.1
**Δ5,23-Stigmastadienol**	13.6 ± 1.6	12.1 ± 0.8	9.3 ± 0.3	7.9 ± 0.5	9.9 ± 1	9.1 ± 1.2	5.0 ± 0.2	5.7 ± 0.7
**β-Sitosterol**	1024.6 ± 16.6	1295.3 ± 12.9	938.1 ± 11.7	723.1 ± 5.4	819.3 ± 21.7	888.5 ± 5.2	569.9 ± 6.8	603.3 ± 3.2
**Sitostanol**	9.3 ± 0.7	7.6 ± 0.4	5.9 ± 0.5	4.9 ± 0.6	7.5 ± 1.2	6.4 ± 0.5	6.2 ± 0.2	6.2 ± 0.9
**Δ5-Avenasterol**	64.0 ± 0.9	100.2 ± 2.8	54.6 ± 8.1	116.7 ± 2.9	85.4 ± 7	105.4 ± 7.2	80.0 ± 2.1	76.5 ± 1.7
**Δ5,24-Stigmastadienol**	6.5 ± 0.7	7.3 ± 0.6	3.9 ± 0.9	4.8 ± 0.4	4.1 ± 1	4.7 ± 0.2	3.4 ± 0.5	3.4 ± 0.3
**Δ7-Stigmastenol**	5.1 ± 0.1	3.6 ± 0.7	2.4 ± 0.2	2.8 ± 0.4	20.1 ± 1.1	1.8 ± 0.4	1.3 ± 0.3	nd
**Δ7-Avenasterol**	5.2 ± 0.7	6.5 ± 0.1	1.6 ± 0.4	7.3 ± 0.3	2.0 ± 0.4	1.1 ± 0.2	1.0 ± 0.2	nd
**Sum**	1535.5 ± 27.4	2217.7 ± 6.2	1435.5 ± 19.9	997.8 ± 10.3	1111.0 ± 32.6	1526.0 ± 19.8	871.3 ± 10.4	887.3 ± 7.2

Results are expressed as mean ± SD (n = 3), nd: not detected

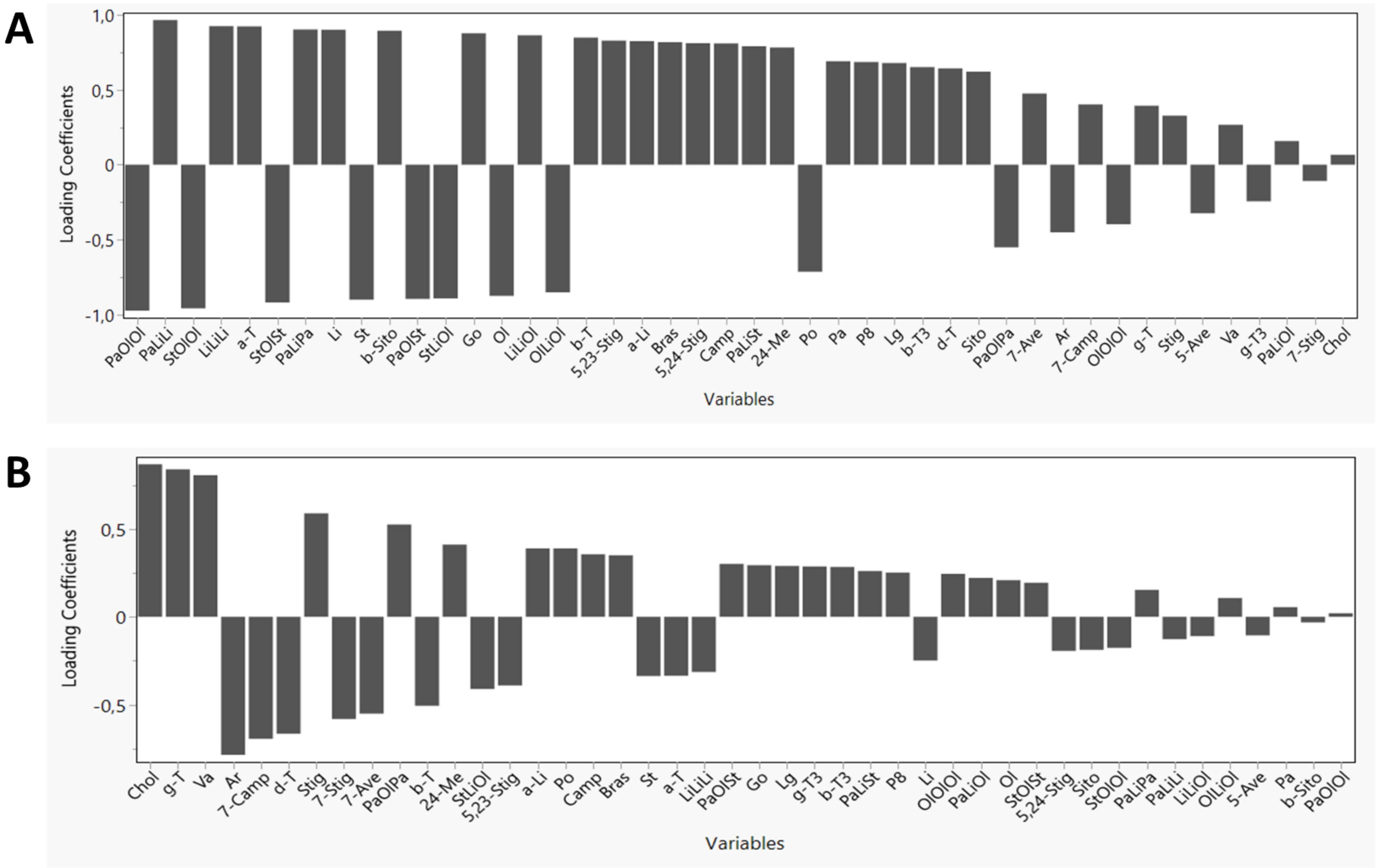

Figure S1. Loadings of the two principal component analysis, A: for PC1 and B: for PC2. Ol: oleic acid, Va: Vaccenic acid, Li: Linoleic acid, a-Li: α-linolenic acid, Ar: arachidic acid, Go: gondoic acid, Lg: lignoceric acid, a-T: α-tocopherol, b-T: β-tocopherol, g-T: γ tocopherol, b-T3: β-tocotrienol, P8: Plastochromanol-8, g-T3: γ-tocotrienol, d-T: δ-tocopherol, Chol: Cholesterol, Bras: brassicasterol, 24-Me: 24-methylenecholesterol, Camp: campesterol, Stig: stigmasterol, 7-Camp: Δ7-campesterol, 5,23-Stig: Δ5,23-stigmastadienol, b-Sito: β-sitosterol, Sito: sitostanol, 5-Ave: Δ5-avenasterol, 5,24-Stig: Δ5,24-stigmastadienol, 7-Stig: Δ7-stigmastenol, 7-Ave: Δ7-avenasterol.
